# Ultraviolet Light Protection: Is It Really Enough?

**DOI:** 10.3390/antiox11081484

**Published:** 2022-07-29

**Authors:** Patricia K. Farris, Giuseppe Valacchi

**Affiliations:** 1 Department of Dermatology, Tulane University School of Medicine, New Orleans, LA 70112, USA; pfarris@tulane.edu; 2 Department of Biomedical and Specialist Surgical Sciences, University of Ferrara, I-44121 Ferrara, Italy; 3 Animal Science Department, Plants for Human Health Institute, NC Research Campus, NC State University, Kannapolis, NC 28081, USA; 4 Department of Food and Nutrition, Kyung Hee University, Hoegi-Dong, Dongdaemun-Gu, Seoul 130-701, Korea

**Keywords:** skin aging, ultraviolet light, solar radiation, pollution, reactive oxygen species, oxidative stress, antioxidants, chelators

## Abstract

Our current understanding of the pathogenesis of skin aging includes the role of ultraviolet light, visible light, infrared, pollution, cigarette smoke and other environmental exposures. The mechanism of action common to these exposures is the disruption of the cellular redox balance by the directly or indirectly increased formation of reactive oxygen species that overwhelm the intrinsic antioxidant defense system, resulting in an oxidative stress condition. Altered redox homeostasis triggers downstream pathways that contribute to tissue oxinflammation (cross-talk between inflammation and altered redox status) and accelerate skin aging. In addition, both ultraviolet light and pollution increase intracellular free iron that catalyzes reactive oxygen species generation via the Fenton reaction. This disruption of iron homeostasis within the cell further promotes oxidative stress and contributes to extrinsic skin aging. More recent studies have demonstrated that iron chelators can be used topically and can enhance the benefits of topically applied antioxidants. Thus, an updated, more comprehensive approach to environmental or atmospheric aging protection should include sun protective measures, broad spectrum sunscreens, antioxidants, chelating agents, and DNA repair enzymes.

## 1. Introduction

The clinical stigmata of skin aging are the result of intrinsic or natural aging superimposed by extrinsic or environmental aging. Dermatologists have long used the term photoaging as a synonym for extrinsic aging, since it is known that exposure to ultraviolet light (UVL) is a major contributor to the phenotype of aging skin [1,2]. More recently, however, they have come to view extrinsic aging differently. Studies have demonstrated that skin is exposed to a variety of internal and external environmental factors that influence the aging process [3,4]. These factors, referred to as the exposome, include not only ultraviolet light but visible light (VL) and infrared (IR), pollution, cigarettes, temperature, poor diet, lack of sleep, and stress [3]. Thus, instead of using photoaging as a synonym for extrinsic aging, the term atmospheric aging is gaining favor, as it acknowledges that there is more than one important environmental factor that accelerates skin aging [5]. In this review, we will discuss the role of light and pollution on skin aging and how to best protect against environmental aggressors, with a focus on the use of topical antioxidants and chelating agents.

## 2. Ultraviolet Light

**Figure 1:**
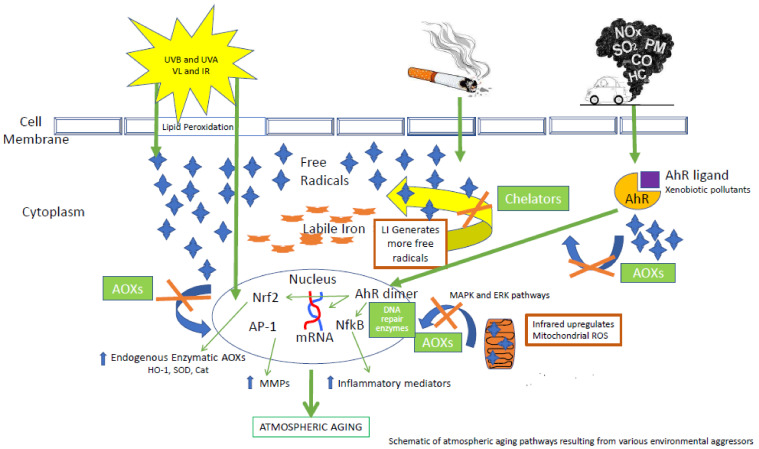
The skin is exposed to a variety of environmental aggressors including solar radiation, cigarette smoke and pollutants that increase directly or indirectly the production of reactive oxygen species (ROS). Exposure to infrared light (IR) upregulates mitochondrial ROS, further increasing intracellular ROS accumulation. In addition, both UV light and pollution increase intracellular free iron, or labile iron (Fe(II)-ion), which further catalyzes formation of ROS. As ROS accumulate within the cell, they overwhelm the intrinsic antioxidant defense system, triggering oxidative stress. Oxidative stress upregulates redox sensitive transcription factors, such as activator protein 1 (AP-1) involved in transcribing for collagen degrading matrix metalloproteinases (MMPs) and nuclear factor kappa beta (NF-кβ), master regulator of the pro-inflammatory cytokines production. Polycyclic aromatic hydrocarbons (PAHs) in particulate matter are ligands of aryl hydrocarbon receptors (AhR). PAHs activate AhR that translocate to the nucleus and after binding to the DNA (xenobiotic-responsive element) induces the transcription of detoxifying enzymes that are able to increase the ROS levels and further activate redox sensitive transcription factors, such as NF-кβ and nuclear factor erythroid 2- related factor 2 (NRF2). Prevention strategies include antioxidants (AOXs) that can be delivered topically or through nutrition to increase the skin’s ability to neutralize ROS. Topically applied chelators can also be used to bind metals, thus preventing ROS generation.

The prolonged exposure to UVL is associated with many skin pathologies, including sunburn, photoaging and skin cancer. Effective strategies to protect against UV-induced skin damage are, therefore, essential. Photoaged skin is characterized by wrinkling, mottled pigmentation, brown spots, scaling, and dryness [6]. In more terminal forms of photoaging, actinic keratoses and skin cancers, including melanoma and non-melanoma skin cancers, occur. Thus, protection from UVL is necessary not only to preserve skin’s appearance, but to improve health span as well (Figure 1).

UVL as it reaches the earth’s surface is 95% ultraviolet A (UVA) and 5% ultraviolet B (UVB). UV light penetrates the skin in a wavelength dependent manner [7]. UVB is a shorter wavelength of light (290–320 nm). It penetrates through the epidermis to the basal layer, where it is absorbed by chromophores including melanin, urocanic acid, porphyrins, nucleic acids, amino acids tryptophan and tyrosine. [5]. Hydrogen peroxide and reactive oxygen species (ROS), such as singlet oxygen and hydroxyl radicals, are generated by UVB’s interaction with chromophores. In addition, UVB has a direct mutagenic effect on DNA, causing the signature mutations cyclobutane pyrimidine dimers (CPDs) and pyrimidine cross-linked dimers [8]. If not repaired, for instance, by nucleotide excision repair (NER) or base excision repair (BER) mechanisms, these mutations found in keratinocytes accumulate, possibly leading to carcinogenesis. UVA is a longer wavelength of light (320–400 nm) and penetrates more deeply into the skin, reaching the dermal layer. UVA is more efficient than UVB at oxidation and exerts its harmful effects on the skin by further generating ROS. It is of interest that the chromophores for UVA remain elusive and only trans-urocanic acid has been identified [9]. UVA causes DNA damage primarily through oxidation of guanine to 8-hydroyx-deoxyguanine (8-OHdg), thus contributing to carcinogenesis [10]. Additionally, UVL exposure suppresses the number and function of Langerhans cells (antigen presenting cells) in the skin. This immunosuppression is a major contributor to photocarcinogenesis [11].

**Scheme 1:**
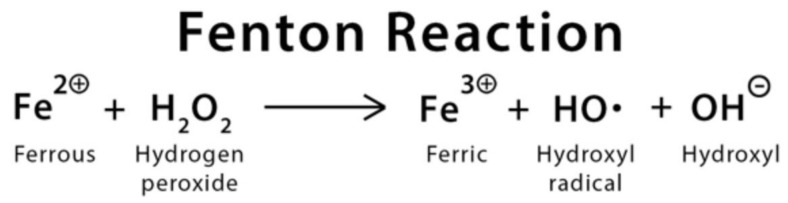
The Fenton reaction describes the formation of hydroxide (OH^−^) and hydroxyl radical by a reaction between iron (II) (Fe^2+^) and hydrogen peroxide (H_2_O_2_).

UVA exposure has a profound effect on the dermal matrix and is the main contributing wavelength to photoaging. Photoaging occurs via oxidative stress triggered by UVA exposure [9]. UVA triggers immediate release of free iron or labile iron (Fe(II)-ion) via proteolysis of ferritin [12]. Free iron catalyzes hydroxyl radical generation via the Fenton reaction (Scheme 1), further contributing to UVA-induced ROS accumulation [13].

Under normal circumstances, endogenous antioxidants, such as vitamin C, vitamin E, carotenoids, ubiquinol, alpha lipoic acid, and urocanic acid, protect skin from UVA-induced ROS by neutralizing them [14,15]. Enzymatic antioxidants, including superoxide dismutase, glutathione peroxidase and hemoxygenase 1, also play a key role in neutralizing free radicals and help to regenerate non-enzymatic antioxidants that have been used [14,15]. These enzymatic antioxidants are under the transcriptional control of nuclear factor erythroid 2-related factor 2 (Nrf2). The Nrf2 signaling pathway is activated in response to oxidative stress and leads to the dissociation from Keap1, allowing its translocation into the nucleus to bind to the antioxidant response element (ARE) [16]. This binding upregulates the transcription of several genes involved in enzymatic antioxidant production, including intracellular heme oxygenase 1, catalase, superoxide dismutase (SOD) and glutathione peroxidase. Oxidative stress ensues when the endogenous antioxidant defense system is overwhelmed.

If left unchecked, ROS induced by UVA exposure can directly damage DNA, cell membranes and dermal matrix proteins, such as collagen and elastin [17]. ROS can also indirectly damage dermal proteins by upregulating redox sensitive transcription factors, such as activator protein 1 (AP-1) and nuclear factor beta (NF-kβ), both important in extracellular matrix homeostasis. The activation of AP-1 increases the production of matrix metalloproteinases (MMPs) that breakdown collagen and elastin [17]. ROS-induced AP-1 activity also downregulates transforming growth factor beta (TGF-β) signaling to fibroblasts, reducing collagen production [18]. It is this net loss in dermal collagen that causes the exaggerated wrinkling observed in actinically damaged skin [18]. In addition, NF-kβ activation leads to premature senescence of keratinocytes, fibroblasts, melanocytes and preadipocytes [19]. These cells display a pro-inflammatory senescence-associated secretory phenotype (SASP) and release inflammatory mediators, including tumor necrosis factor alpha (TNF-α), interleukin 1 (IL-1), IL-6 and IL-8 [20]. This chronic low grade inflammatory state contributes to skin aging in a process now referred to as inflammaging [19,20,21].

## 3. Beyond UV: Visible Light and Infrared

Visible light (VL) is a longer wavelength of light than UV (400–700 nm) and accounts for 50% of solar radiation. Visible light is divided by color and detected by the eye. VL has gained recent interest, as our exposure to high energy blue light (400–500 nm) from devices such as computer screens and smartphones is on the rise. VL from sun exposure is of greater intensity than VL from interior sources, so it contributes more significantly to skin damage. Melanin, water, riboflavin, hemoglobin, and bilirubin are chromophores that are receptive to VL [22]. The role of visible light in the process of skin aging was recently reviewed [23]. In vitro studies demonstrate that cultured fibroblasts exposed to VL generate ROS in a dose-dependent fashion [24]. Wavelengths between 400 and 450 nm resulted in the highest rate of ROS generation, with almost no effect noted at wavelengths greater than 500 nm [24]. Similarly, dose-dependent increases in ROS, IL-1α and MMP-1 were observed in human skin equivalents exposed to VL [25]. In addition, stress-induced changes in the morphology of cultured human fibroblasts were noted following exposure to VL [26]. An increased expression of enzymes involved in collagen degradation, such as MMP-1 and cathepsin K (CTSK), accompanied the morphologic changes observed after VL exposure [26]. Thus, it is likely that visible light contributes to skin aging.

VL appears to play a major role in inducing pigmentation, particularly in melanin-rich skin. It is now known that the key sensor in melanocytes for VL is the opsin 3 receptor [27]. Clinical studies have shown that VL induces erythema in darker skin types [28]. This is likely because there is more melanin in skin of color to absorb VL, inducing heat and causing vasodilation. VL also causes immediate pigment darkening, tanning and long-lasting pigmentation in Fitzpatrick skin types IV-VI, but not in skin type I [28]. Using LEDs on the backs of human subjects, exposure to shorter wavelengths of VL, such as blue light (415 nm), resulted in hyperpigmentation, while red light (630 nm) had no effect [29]. Since the currently available sunscreens provide little protection against VL, alternative protection strategies are necessary.

Infrared (IR) (760 nm–1 mm) makes up almost 50% of the solar energy that reaches the earth’s surface. IR is divided by wavelength into IR-A (760–1400 nm), IR-B (1400–3000 nm) and IR-C (3000 nm–1 mm). IR exposure generates heat energy and can increase the temperature of the skin to more than 40 degrees centigrade [30]. Erythema ab igne, a mottled reticulated erythema, is the result of chronic exposure to heat from space heaters, heating pads and hot water bottles. It is also observed in jewelers, metalworkers, glassblowers, and cooks [31]. Histologically, this condition is characterized by degradation of dermal connective tissue and alteration of elastic tissue, such as the elastotic changes observed in solar elastosis [32]. Heat is known to increase the expression of MMP-12, which can degrade the elastin fiber network. Heat also increases fibrillin-1, 2 and tropoelastin mRNA, further contributing to the deposition of elastotic material [32]. Thus, IR contributes to the accumulation of elastotic material in photoaged skin. IR also induces angiogenesis by altering the balance between vascular endothelial growth factor (VEGF) and endogenous angiogenic inhibitor [33].

It is of interest that IR-A, also called near infrared radiation, penetrates human skin, deeply reaching the level of the subcutaneous tissue without raising skin temperature. IR-B and IR-C increase skin temperature and are absorbed mostly in the epidermis [34]. When exposed to IR, the skin temperature rises to a point and then plateaus. The dose required to reach the plateau is called the minimal heating dose [30]. Studies on human skin irradiated with the minimal heating dose demonstrate that a single dose of IR increases type 1 procollagen expression, while repeated exposures reduce it [35]. This is mediated by changes in the transforming growth factor beta (TGF-β) pathway signaling in response to IR. Repeated IR exposure also increases MMP-1 in the dermis, causing collagen breakdown [35]. Animal studies have confirmed that IR contributes to skin wrinkling. Hairless albino mice irradiated with repeat doses of IRA developed course wrinkling after 15 weeks, while exposure to IRA plus UV irradiation produces wrinkling greater than that achieved by either wavelength alone [36]. Thus, there appears to be an additive effect between UV and IR for inducing skin aging.

Although both IRA and UV cause oxidative stress, IRA plays a role in generating mitochondrial ROS. Copper atoms found in complex IV of the respiratory chain serve as the major IRA chromophore [37]. Upregulation of ROS within mitochondria is believed to initiate IR-mediated damage. These ROS trigger signaling mechanisms start in the mitochondria and diffuse into the cytoplasm, where they increase intracellular calcium levels and activation of extracellular signal-regulated kinase (ERK) and mitogen active protein kinase (MAPK) pathways. This retrograde signaling in human skin fibroblasts affects gene transcription within the nucleus and is central to IRA-induced skin damage [38].

## 4. Environmental Pollutants

The continuous release within the atmosphere of organic and inorganic particulates as particulate matter (PM), gases (carbon monoxide (CO), sulfur dioxide, nitrous oxides (NOx), chlorofluorocarbons (CFCs, etc.) and other volatile biomolecules (VOCs) from industries, cars exhaust, etc., are contributing to the environmental air pollution phenomena that is now considered an important global health issue. According to the World Health Organization (WHO), 4.2 million premature deaths are linked to ambient air pollution and the incidence of cardiovascular, neurodegenerative, pulmonary, and skin diseases associated with exposure to pollution is rising. Among air pollutants, particulate matter (PM), ozone (O_3_) and cigarette smoke (CS) are considered the most dangerous toxic insults for human health, since they can be both inhaled and directly enter through contact with skin. The mechanism of action of these pollutants varies based on their chemical and physical properties, allowing them to interact differently with the skin, and to induce oxidative and inflammatory reactions that affect the skin’s homeostasis (oxinflammation) [39,40]. However, the impact of environmental pollution on health is a very complex field to investigate, considering the individuality of human beings in terms of genome and habits [4,41], and the ability of toxic compounds to be simultaneously released within the lower atmosphere, interacting with each other. Considering that the deterioration of the O_3_ layer is increasing our exposure to ultraviolet radiations, the interaction between toxic compounds with UV light has becoming more common, resulting in a combined and synergistic noxious effect on human skin tissues in terms of skin pathologies, sunburn, cancer, and photoaging [7,42,43].

## 5. Ozone (O_3_)

Among the outdoor pollutants to which living organisms are exposed to daily, tropospheric O_3_ is one of the most noxious for human health [44,45]. O_3_, or trioxygen, is an unstable blue gas with a pungent smell (resembling chlorine bleach) and it is readily perceptible at a concentration of 0.01 ppb but can reach concentrations as high as 0.8 ppm in polluted cities. Due to its triatomic oxygen structure, O_3_ is quite unstable, resulting in a very strong oxidant molecule. The anthropogenic emissions have led to an increase in tropospheric O_3_ concentration (more than 1000 ppb), which is associated not only with lung diseases, such as asthma, COPD, and lung cancer, but also heart disease, inflammatory skin disorders and skin aging [45,46,47]. Exposure to high levels of O_3_ is associated with depletion of antioxidants levels within the skin and the activation of several inflammatory and oxidative pathways [48,49]. Although ozone cannot penetrate skin, it can initiate free radicals by interacting with biomolecules present within the stratum corneum, including lipids, proteins, and DNA, leading to the production of hydroxyl radical (HO^−^), superoxide anion (O^2−^) and hydrogen peroxide (H_2_O_2_) or nonradical species as aldehydes (4-hydroxy-nonenal, 4-HNE) and related 4-HNE protein adducts (PAs) [40,50,51,52]. O_3_ secondary mediators (ROS and 4-HNE protein adducts) can perpetuate the pollutant damage throughout the skin by interacting with keratinocytes and fibroblasts, inducing oxidative stress reactions and lipid peroxidation. The consequent activation of pro-oxidative and pro-inflammatory pathways, such as NRF2, NF-kβ, aryl hydrocarbon receptor (Ahr) and heat shock proteins (HSPs), triggers the release of oxidative and pro-inflammatory mediators, such as IL-8 and COX2, exacerbating various skin pathologies and the inflammaging process [40,48,53,54,55,56,57]. In addition, O_3_ induces the activation of collagen degrading metalloproteinases (MMPs) MMP2 and MMP9 that play a role in photoaging and skin cancer progression [58].

## 6. Particulate Matter (PM)

Particulate matter (PM) is the principal component of air pollution. It is a mixture of solid and liquid particles suspended in the air, including poly-aromatic hydrocarbons (PAHs), metals, inorganic, and organic toxins, which may have an anthropogenic or a natural biogenic origin. These particles originate naturally from volcanoes, forest fires, living vegetation and dust storms, but are also generated by human activities, such as industrial process, burning of fossil fuels, coal combustion, in vehicles, road dust and in cooling systems. Some of these particles can be involved in oxidative reactions that rely on the oxidation of primary gases, such as nitrogen oxides (NOx), sulfur, but also volatile organic compounds (VOCs). The interaction between the particle’s components (NOx, CO and VOCs), mineral dust, black carbon, and sulfur dioxide (SO_2_) with UV light leads to photochemical smog, which is visible and contributes to the deterioration of the stratospheric O_3_ layer, as well as the formation of the ground level O_3_. PM particles exhibit irregular shapes and are classified based on their aerodynamic diameter. The coarse fraction includes particles with a size ranging from 2.5 to 10 µm (PM_2.5_–PM_10_), whereas the fine fraction contains particles ranging from 0.1 and 2.5 µm. All particles that display a diameter less than 0.1 µm are ultrafine particles (UFPs). Fine and ultrafine particles are the most dangerous for human health, since they can be inhaled, deposited deep in the airways, and may even be distributed systemically. Thus, exposure to PM is associated with the development and exacerbation of respiratory diseases, such as asthma and COPD, [59,60,61] and nervous system-related pathologies [62].

The skin is another important target organ for PM. Particles can directly interact and eventually penetrate cutaneous tissue, contributing to increased oxidative stress, activation of inflammatory pathways, DNA damage and skin aging [5,63,64]. Indeed, despite the inability of O_3_ to cross the skin barrier, some PM components, such as polyaromatic carbons (PAHs), can penetrate transdermally or through hair follicles, triggering the production of ROS and lipid peroxidation (4-HNE), leading to apoptosis, DNA and mitochondria damage, activation of pro-inflammatory pathways (NF-kβ, AP1) and the antioxidant response (NRF2) [5,63,64,65,66]. In addition, oxides of nitrogen display an oxidizing effect in skin tissue [67], and their effect on human skin is strictly related to ultrafine PM and black carbon, since they are all emitted during traffic and industries emission [68,69]. Moreover, considering that PM can be inhaled and be released in the bloodstream, it is possible that some particles can reach the skin and affect skin homeostasis from the inside, thus contributing to the damage induced by air pollutant exposure.

## 7. Cigarette Smoke (CS)

CS is one of the most dangerous indoor and outdoor environmental pollutants. It is a complex aerosol composed of a mix containing more than 4700 chemical substances, distributed in a gas phase and a particulate phase, making CS a peculiar pollutant. CS toxicity is mainly associated with the presence of a high level of pro-oxidants, such as free radicals, which can trigger oxidative stress reactions or can lead to secondary oxidative events, such as lipid peroxidation [40,70,71]. Indeed, CS has been estimated to contain 1014 low molecular-weight carbon- and oxygen-centered radicals within the gas phase [71,72], and nitric oxide (NO), up to 500 ppm, which can be oxidized into NO_2_ and participates in oxidative events [73]. Besides the mainstream smoke represented by the combination of inhaled and exhaled smoke while taking a cigarette puff, second-hand smoke or side stream smoke is released into the air directly by a burning cigarette, displaying even more toxic properties than mainstream CS [74]. In addition to the oxidative damage, CS stimulates the release of pro-inflammatory cytokines and consequent epigenetic modifications [75]. For instance, elevated levels of IL-1β, IL-6 and TNF-α have been found in mice exposed to CS for 120 min/day, as well as elevated levels of MMP-1 [76,77].

Cigarette smoke contributes to premature skin aging and wrinkling and to several inflammatory pathologies. These effects are mainly due its ability to induce ROS production within the epidermal layers, lipid peroxidation and up-regulation of MMPs, such as MMP-1 and -3, which contribute to dermal collagen degradation and skin aging [40,78,79,80,81]. Human keratinocytes exposed to CS display decreased wound-healing capacity, increased NRF2 and MMP9 expression levels, as well as an altered epidermal barrier integrity [82]. Moreover, water-soluble PAHs of CS have been demonstrated to induce an oxidative imbalance and increased NADPH oxidase activity within the skin [79,83]. Thus, CS has been correlated with the development of several skin pathologies, fueling both the oxidative and the inflammatory responses of the cutaneous tissue and compromising skin integrity [84,85,86].

## 8. Synergy between Solar Radiation and Pollution

Despite our daily exposure to different pollutants and solar radiation, few studies have investigated the possible interaction between the two and their combined effects on skin. Although UV radiation is the most aggressive environmental agent, the role of pollution in damaging skin is significant. The impact of the combined exposure to pollutants and UV radiations on skin is due to their additive effects on oxinflammation [42,43,87,88,89,90,91]. PM, due to its composition, has been shown to interact with UV light [90]. Poly aromatic hydrocarbons from PM and cigarette smoke can absorb UVA photons, and therefore be photoactivated by UV, exacerbating skin damage. Indeed, photoactivated PAHs can transfer energy and electrons to oxygen (singlet oxygen), initiating oxidative reactions that lead to the production of ROS and DNA damage [91,92,93,94], and ultimately skin damage [95]. Squalene, one of the main lipids present within the skin, can be oxidized by singlet oxygen, suggesting that the combination of UV and PAHs could exacerbate skin damage [96,97,98]. O_3_, in combination with UV light, can potentiate UV-induced depletion of vitamin E, which is essential for skin health [99,100]. The oxinflammatory damage induced by pollutants can then culminate in the alteration of skin functionality by affecting the main components of the stratum corneum (SC), the cornified envelope, which is the primary barrier of the skin [101]. PM, O_3_ and UV are all able to modulate cutaneous proteins that are essential for skin differentiation and proper barrier function, such as involucrin, filaggrin, and keratins [102,103,104], potentiating the damage when acting together [42,105]. Moreover, pollutants can affect other essential skin barrier components, such as tight junctions (TJs), including claudin-1, zonula occludens-1 (ZO-1), occludins and water channels that are involved in maintaining skin barrier integrity [106,107]. Pollutant exposure and UV radiation as well can compromise the distribution of TJs within human skin and keratinocytes, deteriorating the cutaneous tissue functionality [108]. For instance, PM has been found to downregulate ZO-1 via a redox mechanism [109]. These findings suggest that the combination of pollutants can enhance skin damage by acting synergistically to activate inflammatory pathways and to induce oxidative stress reactions, exacerbating skin disorders, carcinogenesis and contributing to the skin inflammaging [5,110,111,112]. Of note, all air pollutants and UV radiation can modulate important inflammatory platforms of the innate immune system, the inflammasomes, via oxidative-related mechanisms [49,113,114,115]. Inflammasomes are multiprotein complexes that form in the cytoplasm and regulate IL-1 cytokines [116]. Nod-like receptors, including NLRP1 and NLRP3, are integral to the formation of inflammasomes and are activated in several skin conditions related also to pollutant exposure [91,116,117].

The transcription factor aryl hydrocarbon receptor (AhR) represents another important target for air pollutants, mediating both the antioxidant and the inflammatory responses. AhR activation has been involved in the exacerbation of skin pathologies such as acne, atopic dermatitis, skin aging, carcinogenesis, and skin barrier impairment [118,119,120,121,122,123]. Air pollutants, such as PAHs in PM, O_3_ but also UV, can mediate the upregulation of CYP1 enzymes and reactive oxygen species (ROS) via AhR receptor activation, exacerbating the skin damage when acting together [42,105,121,124,125,126].

## 9. Environmental Protection Strategies

### 9.1. Sunscreen

As reviewed here, there is a need for broad spectrum protection against solar radiation, including UV, VL and IR. Currently, most broad-spectrum sunscreens provide protection against UVB radiation and shorter wavelength UVA. There is a paucity of FDA-approved sunscreen ingredients that protect against longer wavelength UVA (>370 nm) and none that confer protection against visible light [127]. Thus, it is not surprising that sunscreens touting UVA protection were found to reduce UVA-induced ROS formation by only 55% [128]. Tinted cosmetics and sunscreens that contain iron oxides are now recommended to extend protection into the visible light spectrum [129], but unfortunately do not confer protection against IR. Sunscreens, of course, provide no protection against environmental pollutants; thus, other strategies are necessary to prevent oxinflammatory damage caused by this pollutant exposure (Table 1).

### 9.2. Antioxidants Compounds as a Therapeutic Approach to Prevent the OxInflammatory Damage within the Skin

Our skin is equipped with defense systems to counteract oxidative damage induced by environmental exposure. However, the depletion of skin defensive system can lead to premature skin aging [55]. To counteract oxidative damage, topical antioxidant formulations have been developed to protect skin [130]. Moreover, a healthy diet, including vitamins and micronutrients helps restore the gut microbiome, and provides further protection to preserve the skin health [131].

#### 9.2.1. Endogenous Defensive Enzymes

As mentioned above, human skin contains endogenous enzymes, such as superoxide dismutase (SOD), catalase (Cat) and glutathione peroxidase (GPx), which scavenge ROS and protect our skin from environmental insults [132,133,134]. These enzymes are able to catalyze the dismutation of the superoxide radical O_2_^−^ into oxygen and H_2_O_2_ (SOD) and to convert H_2_O_2_ in water (Cat, GPx), therefore preventing the production of hydroxyl radicals (^.^HO) known to trigger lipid peroxidation [134,135,136]. However, environmental pollutants can modulate the activity of these cutaneous antioxidant enzymes and alterations in their activity can be associated with inflammatory skin conditions [137,138,139], altering skin redox homeostasis, skin barrier function, and lead to skin aging and carcinogenesis [14,140,141,142]. PM exposure has been associated with elevated levels of SOD and GPx in human keratinocytes and higher levels of oxidative stress [143], whereas a decrease in Cat activity was noted in mice skin exposed to UVB radiation [144]. Environmental pollutants have been found to increase the cutaneous levels of SOD and oxidized stratum corneum proteins in patients with atopic dermatitis [145], suggesting that they might be involved in the exacerbation of skin pathologies by modulating the antioxidant response. Nevertheless, the depletion of these enzymes caused by environmental pollutants can be restored by the application of antioxidant compounds [54,146,147,148].

#### 9.2.2. Skin Micronutrients and Topical Antioxidant Application

Besides endogenous enzymes, skin is equipped with a non-enzymatic set of antioxidant molecules that can be absorbed through the diet and they play a key role in protecting the skin from oxidative reactions, and lipid peroxidation, and promote keratinocyte differentiation, as well as skin barrier function [14,149,150,151]. The most abundant antioxidant components within the cutaneous tissue are tocopherol (vitamin E) and ascorbic acid (vitamin C) [152,153]. Carotenoids, uric acid, and co-enzyme Q_10_ (CO-Q_10_) are other important micronutrients with antioxidant properties for human skin [55]. Pollutant-induced photo-oxidative stress can lead to the depletion of skin surface antioxidants, especially vitamin E, vitamin C and glutathione, resulting in structural skin damage and an impairment of the barrier function, as well as skin aging. For instance, O_3_ has been shown to deplete vitamins E and C in mice skin, leading to lipid peroxidation [154,155,156]. The application of topical compounds containing vitamin E, C and caffeic acid can prevent skin structural damage, counteracting photoaging, DNA damage and reducing UV-induced free radical production [150,157,158,159]. Indeed, the photoprotection properties of vitamin C resides in its ability to inhibit the UV-induced activation of AP-1 or NF-kβ, which in turn upregulate MMPs, leading to collagen degradation and consequent wrinkle formation, photoaging and elastin accumulation [160]. Moreover, vitamin C displays an antiaging effect by helping in the cross-linking of collagen fibers, in the biosynthesis of collagen [152], and in reducing oxidized vitamin E [161]. However, L-ascorbic acid, which is the form of vitamin C found in the skin, is a hydrophilic molecule and it displays poor penetration properties through the stratum corneum [162]. To improve L-ascorbic acid stabilization and permeability, new formulations containing other compounds, such as ferulic acid or esterified forms of vitamin C, have been established [163,164]. Indeed, ferulic acid can stabilize vitamin C solutions by adjusting the pH and favoring skin permeability [165]. Several new formulations containing these compounds have been shown to protect from UV-induced photodamage by improving the protecting effect of UV filters [166,167,168], and they can also prevent O_3_ cutaneous damage [146]. Another important skin micronutrient, β-carotene, belongs to the carotenoid family, and is a precursor of vitamin A. β-carotene needs to be taken in through diet and can inhibit the lipoxygenase enzymes that are responsible for the production of ROS. Moreover, β-carotene quenches singlet oxygen and peroxyl radicals and can protect skin from sunlight and photodamage, together with vitamin E [169,170]. Indeed, the chlorophyll present within carotenoids can absorb UVA through the porphyrin-related molecular structure called chlorin [171]. Other plant antioxidants, including epigallocatechin gallate (EGCG), resveratrol, and lycopene, have been shown to protect skin against UV-induced damage [15]. Coenzyme Q10 (CoQ10) or ubiquinone is a co-enzyme involved in metabolic cells processes, such as the production of energy within mitochondria, and displays antioxidant properties [171,172]. Although CoQ10 topical absorption can be limited, its topical application, together with dietary intake, has been demonstrated to improve the antioxidant defense of the skin, prevent wrinkles and skin aging [173,174,175].

Protection against IRA-induced damage by topical antioxidants has also been demonstrated. A randomized, prospective, controlled, double-blind study was conducted to compare the efficacy of an SPF30 sunscreen alone versus the same sunscreen supplemented with an antioxidant mixture for protecting against IRA-induced damage [176]. As expected, IRA exposure upregulated MMP-1 expression compared to unirradiated skin and pretreatment for ten days with an SPF30 sunscreen supplemented with vitamin C, vitamin E, ubiquinone, and grape seed extract that effectively prevented IRA-induced MMP-1 mRNA expression. In contrast, the use of SPF30 sunscreen alone did not provide significant protection. This study confirms that topical antioxidants can be used to mitigate the detrimental effects of IRA on human skin. Similar results have been shown with topical formulations with ferulic acid combined with vitamins C and E [177]. Antioxidants also appear promising for protecting against VL. [24,178] Licochalcone A added to cultured fibroblasts prevented VL-induced upregulation of ROS compared to UV filters alone [24]. More recently, the use of an antioxidant enriched sunscreen was found to reduce VL-induced pigmentation in human skin when compared to the sunscreen alone [178]. The protection was equivalent to that of a tinted sunscreen.

### 9.3. Chelating Agents to Modulate and Iron and Redox Homeostasis in Skin

Besides the utilization of natural antioxidant compounds, another way to counteract ROS production induced by environmental pollutants and UV radiation is the application of chelating agents in cosmetic formulations. Chelating agents can bind with metal ions, such as copper and iron, preventing them from chemically reacting with other substances [179]. Iron has been identified as one of the primary metals modulated by air pollutant exposure in human skin [180,181]. Air pollutants have been shown to lead to perturbation of iron homeostasis, resulting in iron accumulation within cells that can be used as a catalyzing agent in Fenton’s reaction to produce ROS [12,44,45,182,183]. Indeed, iron is an essential cofactor involved in several biological and metabolic functions, such as transport of oxygen, DNA synthesis and electron transport. Excessive amounts of iron within cells can interact with O_2_^•−^, fueling mitochondrial ROS production that can cause oxidation of biomolecules such as lipids, DNA, and proteins [184], resulting in the development of several oxidative stress-related pathologies [185,186,187]. As discussed previously, UVA radiation, as well as other pollutants, such as PM and O_3_, can induce the release of iron from the iron-binding proteins, resulting in the accumulation of labile iron (Fe(II)-ion), within the skin, resulting in ROS production [12,180,181,188,189]. The use of drugs that are able to reduce the excessive accumulation of iron within cells is a novel strategy to prevent iron from participating in Fenton’s reaction and to attenuate lipid peroxidation in response to pollutant exposure. Data suggest that the combination of iron chelators and antioxidant compounds work synergistically to counteract skin photodamage [190,191], representing a good approach to enhance the protective effect of formulations containing antioxidant compounds. Deferoxamine (DFO) is one of the most potent iron chelators with a high affinity for iron and it has been used since 1986 to treat iron overdoses, hemochromatosis, and blood transfusions [192,193]. DFO can help in skin wound healing and diabetic ulcer regeneration by upregulating the hypoxia-inducible factor-1 alpha (HIF-1a) that is involved in angiogenesis, vascularization and in regulating other important mediators, such as the vascular endothelial growth factor (VEGF), which help bring nutrients and other essential factors for tissue regeneration [194,195,196,197]. Besides angiogenesis properties, DFO displays antioxidant properties, due to its ability to directly quench ROS, such as HO and O_2−_, and can form the deferoxamine nitroxide radical (DfNO) [198,199]. Moreover, DFO can stop the iron-mediated propagation of lipid peroxidation by quenching and reducing the levels of alkoxyl and peroxyl radicals [200]. In a study designed to evaluate the protective effects of topically applied DFO, singularly or in combination with antioxidants, human skin explants were exposed to diesel engine exhaust (DEE). DFO alone and in combination with antioxidants vitamin C, E and ferulic acid was effective at counteracting DEE-induced skin damage, including lipid peroxidation, MMP-9 activation, and loss of filaggrin and involucrin [89]. In a recent review on the role of iron and redox homeostasis in skin aging, the authors suggest the need for studies to identify botanicals that have both antioxidant and iron chelating properties [201]. These naturals would be bi-functional ingredients capable of modulating iron and redox homeostasis and provide skin benefits, including environmental protection and anti-aging. Although, out of the scopes of this review, it should be mentioned that several natural compounds, including polyphenols, anthocyanins, and flavonoids, have shown to protect the skin against outdoor stressors, as reviewed elsewhere [202].

**Table 1:** Cutaneous stressors, skin damage and protection technologies.

Environmental Aggressor	Solar Radiation		Pollution			
	UVA/UVB	VL and IR	Tropospheric Ozone (O_3_)	Particulate Matter	Transition Metals	Cigarette Smoke
** Biomarkers of Exposure and Damage **	**UVB (290–320 nm)** [5,8] Skin erythema DNA mutations (CPDs) **UVA (320–400 nm)** [11,12,17,18] Dermal matrix protein degradation ✓ Reduction in collagen and elastin via upregulation of MMPs ✓ Reduction in collagen production via downregulation of TGFβ pathway Release of labile iron (Fe(II)-ion) Suppression of cutaneous APC Photodamage	**VL (400–700 mm)** [23,24,25,26,27,28,29] Includes high energy blue light (400–500 nm) Erythema in darker skin types Immediate and long-lasting pigmentation in darker skin types **Increase** MMPs Increase inflammatory cytokines **IR (760 nm–1 mm)** [30,31,32,33,34,35,36] Generates heat energy and increases skin temperature Erythema ab igne Increase MMPs Accumulation of elastotic material Induces angiogenesis ROS within mitochondria Modulates fibroblast activity	Interacts with stratum corneum lipids, proteins, and DNA Increases ROS HO^−^, O^2−^, and H_2_O_2_ [40,50,51,52] 4-HNE adducts form and induce further lipid peroxidation Activation of NF-кβ aryl hydrocarbon receptor Ahr and HSPs enhance release of IL-8, COX_2_ (ozone-induced inflammation) [40,48,53,54,55,56,57] Increases in MMP-2 and MMP-9 (ozone-induced aging) and skin cancer progression, respectively [58]	**Particulate matter (PM)** Includes poly-aromatic hydrocarbons (PAHs), metals, inorganic and organic toxins Combines with oxidized nitrogen oxides, sulfur and volatile organic compounds and UV light to form photochemical smog Decreases stratospheric O_3_ Increases ground level O_3_ Absorbed through inhalation, skin and hair 4-HNE from lipid peroxidation can damage DNA and mitochondria Stimulates key oxidative and inflammatory transcription factors NF-кβ, AP-1, NRF2, Ahr [5,63,64,65,66] Increase in skin barrier disruption, inflammatory and skin diseases (COPD, asthma, acne, atopic dermatitis, skin aging) [59,60,61,62]	PM in combination with UV can liberate bound iron in the skin as labile iron Interacts with ROS to cause oxidative stress and damage to mitochondria, lipids, DNA, and cellular proteins [91,92,93,94,95] Increase **lipid peroxidation** Increase MMP-9 Decrease filaggrin and involucrin [102,103,104] Disruption of skin barrier [106,107,108]	Increase ROS Secondary lipid peroxidation Increase release of inflammatory cytokines IL-1α, IL-6. TNF-α Increase MMP-1 Increase collagen degradation Increase cutaneous inflammation and skin diseases [40,78,79,80,81] Increase NRF2 and MMP-9 Decrease skin barrier and wound-healing [82]
** Protection **	**Physical Barriers-** Sun shirts Hats Sunglasses **Sunscreens-** Broad spectrum chemical Mineral **TopicalAOX-** [151,152,153,165,166,167,168] Vitamins C, E Ferulic acid Resveratrol Carotenoids Epigallocatechin gallate (EGCG) Co-enzyme Q_10_ Caffeic acid Lycopene Other botanicals **Chelators** [190,191] 2-furildioxime Kojic acid **DNA Repair Enzymes with sunscreen and/or AOX** [203,204,205,206,207,208] **Diet, Micronutrients** [131]	**Physical Barriers-** Sun shirts Hats Sunglasses **Sunscreens and Cosmetics-** Tinted containing **iron oxide (FeO) for VL** [129] **Topical AOXs for IRA-** [176,177] Vitamins C, E Ferulic acid Resveratrol Co-enzyme Q_10_ Grape seed extract **Sunscreen with AOX blend for VL** [178] **Diet and Micronutrients** [131]	**Topical AOX-** [42,146,147] Vitamins C, E Ferulic acid Other botanicals **Diet and Micronutrients** [131]	**Topical AOX-** [42] Vitamins C, E Ferulic acid Other botanicals **Diet and Micronutrients** [131]	**Combination-** [89] Chelators PLUS topical AOX **Diet and Micronutrients** [131]	**Topical AOX-** [148] Resveratrol **Diet and Micronutrients** [131]

### 9.4. DNA Repair Enzymes

More recently, DNA repair enzymes, including photolyase and T4-bacteriophase endonuclease V (T4 endonuclease V), have been studied alone or in combination with UVA/UVB sunscreens with or without antioxidants as a supplement to intrinsic DNA repair mechanisms [203,204,205,206,207]. DNA repair enzymes have been shown to scan and repair DNA mutations to prevent the effects of UV damage from skin photoaging to more malignant manifestations, including AK and skin cancers. Beyond prevention, topical application has demonstrated reversal of existing AK lesions and photoaging [208]. In addition to DNA repair, MMP-9 induction and cutaneous inflammatory cytokines have also been shown to be attenuated. Moreover, these beneficial effects occurred with little to no adverse reactions or events reported. While the current formulations are promising, more clinical studies are needed to better understand the full potential of benefits that these enzymes can offer in mitigating the effects of UV and other environmental aggressors.

## 10. Conclusions

While UV light has long been viewed as the primary offender of inducing environmental cutaneous damage, nowadays, we need to also consider the role of other factors, particularly pollutants, in contributing to skin damage. It is now well documented that stressors such as ozone, particulate matter, cigarette smoke, etc., all components of the so-defined “skin exposome”, can seriously affect cutaneous extrinsic aging and even have an additive effect when present in combination. Therefore, the next generation of cutaneous protection must include not only sunscreen technologies and the usage of hats and clothing, but also new approaches aimed to protect the skin from other stressor sources derived from the pollutants. Indeed, the development of new sunscreens with enhanced UV coverage, DNA repair enzymes, topical antioxidants, and safe and effective chelating agents could better protect our cutaneous tissue from extrinsic aging and pathologies correlated to pollution exposure. This combination approach is necessary to ensure skin health and maintain its youthful appearance.

## References

[1] Kligman L.H., Kligman A.M. (1986). The nature of photoaging: Its prevention and repair. Photodermatology.

[2] Krutmann J., Gilchrest B.A., Gilchrest B.A., Krutmann J. (2006). Photoaging of skin. Skin Aging.

[3] Krutmann J., Bouloc A., Sore G., Bernard B.A., Passeron T. (2017). The skin aging exposome. J. Dermatol. Sci..

[4] Krutmann J., Schikowski T., Morita A., Berneburg M. (2021). Environmentally-Induced (Extrinsic) Skin Aging: Exposomal Factors and Underlying Mechanisms. J. Investig. Dermatol..

[5] McDaniel D., Farris P., Valacchi G. (2018). Atmospheric skin aging-Contributors and inhibitors. J. Cosmet. Dermatol..

[6] Yaar M., Gilchrest B.A., Krutmann J. (2006). Clinical and histological features of intrinsic versus extrinsic skin aging. Skin Aging.

[7] D’Orazio J., Jarrett S., Amaro-Ortiz A., Scott T. (2013). UV radiation, and the skin. Int. J. Mol Sci..

[8] Cadet J., Douki T. (2018). Formation of UV-induced DNA damage contributing to skin cancer development. Photochem. Photobiol. Sci..

[9] Trautinger F. (2001). Mechanisms of photodamage of the skin and its functional consequences for skin ageing. Clin. Exp. Dermatol..

[10] Kielbassa C., Roza L., Epe B. (1997). Wavelength dependence of oxidative DNA damage induced by UV and visible light. Carcinogenesis.

[11] Nishigori C., Yarosh D.B., Donawho C., Kripke M.L. (1996). The immune system in ultraviolet carcinogenesis. J. Investig. Dermatol. Symp. Proc..

[12] Kitazawa M., Iwasaki K., Sakamoto K. (2006). Iron chelators may help prevent photoaging. J. Cosmet. Dermatol..

[13] Pourzand C., Watkin R.D., Brown J.E., Tyrrell R.M. (1999). Ultraviolet A radiation induces immediate release of iron in human primary skin fibroblasts: The role of ferritin. Proc. Natl. Acad. Sci. USA.

[14] Rinnerthaler M., Bischof J., Streubel M.K., Trost A., Richter K. (2015). Oxidative Stress in Aging Human Skin. Biomolecules.

[15] Masaki H. (2010). Role of antioxidants in the skin: Anti-aging effects. J. Dermatol. Sci..

[16] Ryšavá A., Vostálová J., Svobodová A.R. (2021). Effect of ultraviolet radiation on the Nrf2 signaling pathway in skin cells. Int. J. Radiat. Biol..

[17] Fisher G.J., Kang S., Varani J., Bata-Csorgo Z., Wan Y., Datta S., Voorhees J.J. (2002). Mechanisms of Photoaging and Chronological Skin Aging. Arch. Dermatol..

[18] Fisher G.J., Voorhees J.J. (1998). Molecular Mechanisms of Photoaging and its Prevention by Retinoic Acid: Ultraviolet Irradiation Induces MAP Kinase Signal Transduction Cascades that Induce Ap-1-Regulated Matrix Metalloproteinases that Degrade Human Skin In Vivo. J. Investig. Dermatol..

[19] Salminen A., Kaarniranta K., Kauppinen A. (2022). Photoaging: UV radiation-induced inflammation and immunosuppression accelerate the aging process in the skin. Inflamm Res..

[20] Senfetleben U., Karin M. (2002). The IKK/NF-kappaB pathway. Crit. Care Med..

[21] Pilkington S.M., Bulfone-Paus S., Griffiths C.E., Watson R.E. (2021). Inflammaging and the Skin. J. Investig. Dermatol..

[22] Mahmoud B.H., Hexsel C.L., Hamzavi I.H., Lim H.W. (2008). Effects of Visible Light on the Skin. Photochem. Photobiol..

[23] Pourang A., Tisack A., Ezekwe N., Torres A.E., Kohli I., Hamzavi I.H., Lim H.W. (2021). Effects of visible light on mechanisms of skin photoaging. Photodermatol. Photoimmunol. Photomed..

[24] Mann T., Eggers K., Rippke F., Tesch M., Buerger A., Darvin M.E., Schanzer S., Meinke M.C., Lademann J., Kolbe L. (2020). High-energy visible light at ambient doses and intensities induces oxidative stress of skin—Protective effects of the antioxidant and Nrf2 inducer Licochalcone A in vitro and in vivo. Photodermatol. Photoimmunol. Photomed..

[25] Liebel F., Kaur S., Ruvolo E., Kollias N., Southall M.D. (2012). Irradiation of Skin with Visible Light Induces Reactive Oxygen Species and Matrix-Degrading Enzymes. J. Investig. Dermatol..

[26] Zamarrón A., Lorrio S., González S., Juarranz A. (2018). Fernblock Prevents Dermal Cell Damage Induced by Visible and Infrared A Radiation. Int. J. Mol. Sci..

[27] Regazzetti C., Sormani L., Debayle D., Bernerd F., Tulic M., De Donatis G., Chignon-Sicard B., Rocchi S., Passeron T. (2018). Melanocyts sense blue light and regulate pigmentation through opsin-3. J. Investig. Dermatol..

[28] Mahmoud B.H., Ruvolo E., Hexsel C.L., Liu Y., Owen M.R., Kollias N., Lim H.W., Hamzavi I.H. (2010). Impact of Long-Wavelength UVA and Visible Light on Melanocompetent Skin. J. Investig. Dermatol..

[29] Dutiel L., Cardot-Leccia N., Queille-Roussel C., Maubert Y., Harmelin Y., Boukari F., Ambrosetti D., Lacour J.P., Passeron T. (2014). Differences in visible light-induced pigmentation according to wavelengths; a clinical and histological study in comparison to UVB exposure. Pigment Cell Melanoma Res..

[30] Lee H.S., Lee D.H., Cho S., Chung J.H. (2006). Minimal heating dose: A novel biological unit to measure infrared irradiation. Photodermatol. Photoimmunol. Photomed..

[31] Cho S., Shin M.H., Kim Y.K., Seo J.-E., Lee Y.M., Park C.-H., Chung J.H. (2009). Effects of Infrared Radiation and Heat on Human Skin Aging in vivo. J. Investig. Dermatol. Symp. Proc..

[32] Chen Z., Seo J.Y., Kim Y.K., Lee S.R., Kim K.H., Cho K.H., Eun H.C., Chung J.H. (2005). Heat modulation of tropoelastin, fibrillin-1 and matrix metalloproteinase-12 in human skin in vivo. J. Investig. Dermatol..

[33] Kim M.-S., Kim Y., Cho K., Chung J. (2006). Infrared exposure induces an angiogenic switch in human skin that is partially mediated by heat. Br. J. Dermatol..

[34] Schieke S.M., Schroeder P., Krutmann J. (2003). Cutaneous effects of infrared radiation: From clinical observations to molecular response mechanisms. Photodermatol. Photoimmunol. Photomed..

[35] Kim M.-S., Kim Y.K., Cho K.H., Chung J.H. (2006). Regulation of type I procollagen and MMP-1 expression after single or repeated exposure to infrared radiation in human skin. Mech. Ageing Dev..

[36] Kim H.H., Lee M.J., Lee S.R., Kim K.H., Cho K.H., Eun H.C., Chung J.H. (2005). Augmentation of UV-induced skin wrinkling by infrared irradiation in hairless mice. Mech. Ageing Dev..

[37] Krutmann J., Schroeder P. (2009). Role of Mitochondria in Photoaging of Human Skin: The Defective Powerhouse Model. J. Investig. Dermatol. Symp. Proc..

[38] Shroeder P., Calles C., Benesova T., Macaluso F., Krutmann J. (2010). Photoprotection beyond ultraviolet radiation-effective sun protection must include protection against infrared A radiation-induced skin damage. Skin Pharmacol. Physiol..

[39] Valacchi G., Sticozzi C., Pecorelli A., Cervellati F., Cervellati C., Maioli E. (2012). Cutaneous responses to environmental stressors. Ann. N. Y. Acad. Sci..

[40] Pecorelli A., Woodby B., Prieux R., Valacchi G. (2019). Involvement of 4-hydroxy-2-nonenal in pollution-induced skin damage. BioFactors.

[41] Wild C.P. (2005). Complementing the Genome with an “Exposome”: The Outstanding Challenge of Environmental Exposure Measurement in Molecular Epidemiology. Cancer Epidemiol. Biomark. Prev..

[42] Ferrara F., Woodby B., Pecorelli A., Schiavone M.L., Pambianchi E., Messano N., Therrien J.-P., Choudhary H., Valacchi G. (2020). Additive effect of combined pollutants to UV induced skin OxInflammation damage. Evaluating the protective topical application of a cosmeceutical mixture formulation. Redox Biol..

[43] Marrot L. (2018). Pollution and Sun Exposure: A Deleterious Synergy. Mechanisms and Opportunities for Skin Protection. Curr. Med. Chem..

[44] Cross C.E., Valacchi G., Schock B., Wilson M., Weber S., Eiserich J., van der Vliet A. (2002). Environmental Oxidant Pollutant Effects on Biologic Systems: A focus on micronutrient antioxidant—oxidant interactions. Am. J. Respir. Crit. Care Med..

[45] Zhang J., Wei Y., Fang Z. (2019). Ozone Pollution: A Major Health Hazard Worldwide. Front. Immunol..

[46] Mumby S., Chung K.F., Adcock I.M. (2019). Transcriptional Effects of Ozone and Impact on Airway Inflammation. Front. Immunol..

[47] Han M.-H., Yi H.-J., Kim Y.-S., Ko Y., Kim Y.-S. (2016). Association between Diurnal Variation of Ozone Concentration and Stroke Occurrence: 24-Hour Time Series Study. PLoS ONE.

[48] Valacchi G., Pagnin E., Corbacho A.M., Olano E., David P.A., Packer L., Cross C.E. (2004). In vivo ozone exposure induces antioxidant/stress-related responses in murine lung and skin. Free Radic. Biol. Med..

[49] Ferrara F., Pambianchi E., Pecorelli A., Woodby B., Messano N., Therrien J.-P., Lila M.A., Valacchi G. (2019). Redox regulation of cutaneous inflammasome by ozone exposure. Free Radic. Biol. Med..

[50] Ayala A., Muñoz M.F., Argüelles S. (2014). Lipid peroxidation: Production, metabolism, and signaling mechanisms of malondialdehyde and 4-hydroxy-2-nonenal. Oxidative Med. Cell. Longev..

[51] Breitzig M., Bhimineni C., Lockey R., Kolliputi N. (2016). 4-Hydroxy-2-nonenal: A critical target in oxidative stress?. Am. J. Physiol. Cell Physiol..

[52] Niki E. (2015). Lipid oxidation in the skin. Free Radic. Res..

[53] Zhuang Y., Lyga J. (2014). Inflammaging in skin and other tissues—The roles of complement system and macrophage. Inflamm. Allergy-Drug Targets.

[54] Valacchi G., van der Vliet A., Schock B.C., Okamoto T., Obermuller-Jevic U., Cross C.E., Packer L. (2002). Ozone exposure activates oxidative stress responses in murine skin. Toxicology.

[55] Woodby B., Penta K., Pecorelli A., Lila M.A., Valacchi G. (2020). Skin Health from the Inside Out. Annu. Rev. Food Sci. Technol..

[56] Valacchi G., Fortino V., Bocci V. (2005). The dual action of ozone on the skin. Br. J. Dermatol..

[57] Muresan X.M., Sticozzi C., Belmonte G., Savelli V., Evelson P., Valacchi G. (2018). Modulation of cutaneous scavenger receptor B1 levels by exogenous stressors impairs “in vitro” wound closure. Mech. Ageing Dev..

[58] Pittayapruek P., Meephansan J., Prapapan O., Komine M., Ohtsuki M. (2016). Role of Matrix Metalloproteinases in Photoaging and Photocarcinogenesis. Int. J. Mol. Sci..

[59] Leikauf G.D., Kim S.-H., Jang A.-S. (2020). Mechanisms of ultrafine particle-induced respiratory health effects. Exp. Mol. Med..

[60] Hopke P.K., Croft D., Zhang W., Lin S., Masiol M., Squizzato S., Thurston S.W., van Wijngaarden E., Utell M.J., Rich D.Q. (2019). Changes in the acute response of respiratory diseases to PM 2.5 in New York State from 2005 to 2016. Sci. Total Environ..

[61] Doiron D., De Hoogh K., Probst-Hensch N., Fortier I., Cai Y., De Matteis S., Hansell A.L. (2019). Air pollution, lung function and COPD: Results from the population-based UK Biobank study. Eur. Respir. J..

[62] Wang Y., Xiong L., Tang M. (2017). Toxicity of inhaled particulate matter on the central nervous system: Neuroinflammation, neuropsychological effects and neurodegenerative disease. J. Appl. Toxicol..

[63] Magnani N.D., Muresan X.M., Belmonte G., Cervellati F., Sticozzi C., Pecorelli A., Miracco C., Marchini T., Evelson P.A., Valacchi G. (2016). Skin Damage Mechanisms Related to Airborne Particulate Matter Exposure. Toxicol. Sci..

[64] Schikowski T., Hüls A. (2020). Air Pollution and Skin Aging. Curr. Environ. Health Rep..

[65] Piao M.J., Ahn M.J., Kang K.A., Ryu Y.S., Hyun Y.J., Shilnikova K., Zhen A.X., Jeong J.W., Choi Y.H., Kang H.K. (2018). Particulate matter 2.5 damages skin cells by inducing oxidative stress, subcellular organelle dysfunction, and apoptosis. Arch. Toxicol..

[66] Kim M., Kim J.H., Jeong G.J., Park K.Y., Lee M.-K., Seo S.J. (2019). Particulate matter induces pro-inflammatory cytokines via phosphorylation of p38 MAPK possibly leading to dermal inflammaging. Exp. Dermatol..

[67] Bruch-Gerharz D., Ruzicka T., Kolb-Bachofen V. (1998). Nitric oxide in human skin: Current status and future prospects. J. Investig. Dermatol..

[68] Hüls A., Vierkötter A., Gao W., Krämer U., Yang Y., Ding A., Stolz S., Matsui M., Kan H., Wang S. (2016). Traffic-Related Air Pollution Contributes to Development of Facial Lentigines: Further Epidemiological Evidence from Caucasians and Asians. J. Investig. Dermatol..

[69] Levy I., Mihele C., Lu G., Narayan J., Brook J.R. (2014). Evaluating Multipollutant Exposure and Urban Air Quality: Pollutant Interrelationships, Neighborhood Variability, and Nitrogen Dioxide as a Proxy Pollutant. Environ. Health Perspect..

[70] Valavanidis A., Vlachogianni T., Fiotakis K. (2009). Tobacco Smoke: Involvement of Reactive Oxygen Species and Stable Free Radicals in Mechanisms of Oxidative Damage, Carcinogenesis and Synergistic Effects with Other Respirable Particles. Int. J. Environ. Res. Public Health.

[71] Church D.F., Pryor W.A. (1985). Free-radical chemistry of cigarette smoke and its toxicological implications. Environ. Health Perspect..

[72] Jenkins R.A., Tomkins B., Guerin M.R. (2000). The Chemistry of Environmental Tobacco Smoke: Composition and Measurement.

[73] Eiserich J., Vossen V., O’Neill C.A., Halliwell B., Cross C.E., Van Der Vliet A. (1994). Molecular mechanisms of damage by excess nitrogen oxides: Nitration of tyrosine by gas-phase cigarette smoke. FEBS Lett..

[74] Schick S., Glantz S. (2005). Philip Morris toxicological experiments with fresh sidestream smoke: More toxic than mainstream smoke. Tob. Control.

[75] Zong D., Liu X., Li J., Ouyang R., Chen P. (2019). The role of cigarette smoke-induced epigenetic alterations in inflammation. Epigenetics Chromatin.

[76] Zhang J., Liu Y., Shi J., Larson D.F., Watson R.R. (2002). Side-Stream Cigarette Smoke Induces Dose–Response in Systemic Inflammatory Cytokine Production and Oxidative Stress. Exp. Biol. Med..

[77] Yuan H., Wong L.S., Bhattacharya M., Ma C., Zafarani M., Yao M., Schneider M., Pitas R.E., Martins-Green M. (2007). The effects of second-hand smoke on biological processes important in atherogenesis. BMC Cardiovasc. Disord..

[78] Morita A., Torii K., Maeda A., Yamaguchi Y. (2009). Molecular Basis of Tobacco Smoke-Induced Premature Skin Aging. J. Investig. Dermatol. Symp. Proc..

[79] Sticozzi C., Belmonte G., Pecorelli A., Arezzini B., Gardi C., Maioli E., Miracco C., Toscano M., Forman H.J., Valacchi G. (2012). Cigarette Smoke Affects Keratinocytes SRB1 Expression and Localization via H_2_O_2_ Production and HNE Protein Adducts Formation. PLoS ONE.

[80] Just M., Ribera M., Monso E., Lorenzo J., Ferrándiz C. (2007). Effect of smoking on skin elastic fibres: Morphometric and immunohistochemical analysis. Br. J. Dermatol..

[81] Yin L., Morita A., Tsuji T. (2000). Alterations of extracellular matrix induced by tobacco smoke extract. Arch. Dermatol. Res.

[82] Rajagopalan P., Nanjappa V., Raja R., Jain A.P., Mangalaparthi K.K., Sathe G.J., Babu N., Patel K., Cavusoglu N., Soeur J. (2016). How Does Chronic Cigarette Smoke Exposure Affect Human Skin? A Global Proteomics Study in Primary Human Keratinocytes. OMICS A J. Integr. Biol..

[83] Sticozzi C., Belmonte G., Cervellati F., Muresan X., Pessina F., Lim Y., Forman H., Valacchi G. (2014). Resveratrol protects SR-B1 levels in keratinocytes exposed to cigarette smoke. Free Radic. Biol. Med..

[84] Kavli G., Forde O.H., Arnesen E., E Stenvold S. (1985). Psoriasis: Familial predisposition and environmental factors. BMJ.

[85] Naldi L., Peli L., Parazzini F. (1999). Association of early-stage psoriasis with smoking and male alcohol consumption: Evidence from an Italian case-control study. Arch. Dermatol..

[86] Naldi L. (2016). Psoriasis: Targets and Therapy Dovepress Psoriasis and smoking: Links and risks. Psoriasis Targets Ther..

[87] Burke K., Wei H. (2009). Synergistic damage by UVA radiation and pollutants. Toxicol. Ind. Health.

[88] Ali A., Khan H., Bahadar R., Riaz A., Bin Asad M.H.H. (2020). The impact of airborne pollution and exposure to solar ultraviolet radiation on skin: Mechanistic and physiological insight. Environ. Sci. Pollut. Res..

[89] Pambianchi E., Ferrara F., Pecorelli A., Benedusi M., Choudhary H., Therrien J.-P., Valacchi G. (2021). Deferoxamine Treatment Improves Antioxidant Cosmeceutical Formulation Protection against Cutaneous Diesel Engine Exhaust Exposure. Antioxidants.

[90] Mokrzyński K., Krzysztyńska-Kuleta O., Zawrotniak M., Sarna M., Sarna T. (2021). Fine Particulate Matter-Induced Oxidative Stress Mediated by UVA-Visible Light Leads to Keratinocyte Damage. Int. J. Mol. Sci..

[91] Toyooka T., Ibuki Y. (2007). DNA damage induced by coexposure to PAHs and light. Environ. Toxicol. Pharmacol..

[92] Bao L., Xu A., Tong L., Chen S., Zhu L., Zhao Y., Zhao G., Jiang E., Wang J., Wu L. (2009). Activated Toxicity of Diesel Particulate Extract by Ultraviolet A Radiation in Mammalian Cells: Role of Singlet Oxygen. Environ. Heath Perspect..

[93] Wang Y., Gao D., Atencio D.P., Perez E., Saladi R., Moore J., Guevara D., Rosenstein B.S., Lebwohl M., Wei H. (2005). Combined subcarcinogenic benzo[a]pyrene and UVA synergistically caused high tumor incidence and mutations in H-ras gene, but notp53, in SKH-1 hairless mouse skin. Int. J. Cancer.

[94] Teranishi M., Toyooka T., Ohura T., Masuda S., Ibuki Y. (2010). Benzo[a]pyrene exposed to solar-simulated light inhibits apoptosis and augments carcinogenicity. Chem. Interactions.

[95] Soeur J., Belaïdi J.-P., Chollet C., Denat L., Dimitrov A., Jones C., Perez P., Zanini M., Zobiri O., Mezzache S. (2017). Photo-pollution stress in skin: Traces of pollutants (PAH and particulate matter) impair redox homeostasis in keratinocytes exposed to UVA1. J. Dermatol. Sci..

[96] Ryu A., Arakane K., Koide C., Arai H., Nagano T. (2009). Squalene as a Target Molecule in Skin Hyperpigmentation Caused by Singlet Oxygen. Biol. Pharm. Bull..

[97] Boussouira B., Pham D.M. (2016). Squalene and Skin Barrier Function: From Molecular Target to Biomarker of Environmental Exposure. Skin Stress Response Pathways.

[98] Kostyuk V., Potapovich A., Stancato A., De Luca C., Lulli D., Pastore S., Korkina L. (2012). Photo-Oxidation Products of Skin Surface Squalene Mediate Metabolic and Inflammatory Responses to Solar UV in Human Keratinocytes. PLoS ONE.

[99] Valacchi G., Weber S.U., Luu C., Cross C.E., Packer L. (2000). Ozone potentiates vitamin E depletion by ultraviolet radiation in the murine stratum corneum. FEBS Lett..

[100] Packer L., Valacchi G. (2002). Antioxidants and the Response of Skin to Oxidative Stress: Vitamin E as a Key Indicator. Ski. Pharmacol. Physiol..

[101] Rembiesa J., Ruzgas T., Engblom J., Holefors A. (2018). The Impact of Pollution on Skin and Proper Efficacy Testing for Anti-Pollution Claims. Cosmetics.

[102] Moravcová M., Libra A., Dvořáková J., Víšková A., Muthny T., Velebný V., Kubala L. (2013). Modulation of keratin 1, 10 and involucrin expression as part of the complex response of the human keratinocyte cell line HaCaT to ultraviolet radiation. Interdiscip. Toxicol..

[103] Lee C.-W., Lin Z.-C., Hu S.C.-S., Chiang Y.-C., Hsu L.-F., Lin Y.-C., Lee I.-T., Tsai M.-H., Fang J.-Y. (2016). Urban particulate matter down-regulates filaggrin via COX2 expression/PGE2 production leading to skin barrier dysfunction. Sci. Rep..

[104] Hieda D.S., Carvalho L.A.D.C., de Mello B.V., de Oliveira E.A., de Assis S.R., Wu J., Du-Thumm L., da Silva C.L.V., Roubicek D.A., Maria-Engler S.S. (2020). Air Particulate Matter Induces Skin Barrier Dysfunction and Water Transport Alteration on a Reconstructed Human Epidermis Model. J. Investig. Dermatol..

[105] Ferrara F., Pambianchi E., Woodby B., Messano N., Therrien J.-P., Pecorelli A., Canella R., Valacchi G. (2021). Evaluating the effect of ozone in UV induced skin damage. Toxicol. Lett..

[106] Yokouchi M., Kubo A. (2018). Maintenance of tight junction barrier integrity in cell turnover and skin diseases. Exp. Dermatol..

[107] Balda M.S., Matter K. (2014). Epidermal tight junctions in health and disease. Semin. Cell Dev. Biol..

[108] Yuki T., Hachiya A., Kusaka A., Sriwiriyanont P., Visscher M.O., Morita K., Muto M., Miyachi Y., Sugiyama Y., Inoue S. (2011). Characterization of tight junctions and their disruption by UVB in human and cultured keratinocytes. J. Investig. Dermatol..

[109] Wang T., Wang L., Moreno-Vinasco L., Lang G.D., Siegler J.H., Mathew B., Usatyuk P.V., Samet J.M., Geyh A.S., Breysse P.N. (2012). Particulate matter air pollution disrupts endothelial cell barrier via calpain-mediated tight junction protein degradation. Part. Fibre Toxicol..

[110] Franceschi C., Garagnani P., Parini P., Giuliani C., Santoro A. (2018). Inflammaging: A new immune–metabolic viewpoint for age-related diseases. Nat. Rev. Endocrinol..

[111] Man M.Q., Elias P.M. (2019). Could inflammaging and its sequelae be prevented or mitigated?. Clin. Interv. Aging.

[112] Zegarska B., Pietkun K., Zegarski W., Bolibok P., Wiśniewski M., Roszek K., Czarnecka J., Nowacki M. (2017). Air pollution, UV irradiation and skin carcinogenesis: What we know, where we stand and what is likely to happen in the future?. Adv. Dermatol. Allergol..

[113] Fenini G., Grossi S., Contassot E., Biedermann T., Reichmann E., French L.E., Beer H.-D. (2018). Genome Editing of Human Primary Keratinocytes by CRISPR/Cas9 Reveals an Essential Role of the NLRP1 Inflammasome in UVB Sensing. J. Investig. Dermatol..

[114] Ferrara F., Prieux R., Woodby B., Valacchi G. (2021). Inflammasome Activation in Pollution-Induced Skin Conditions. Plast. Reconstr. Surg..

[115] Prieux R., Ferrara F., Cervellati F., Guiotto A., Benedusi M., Valacchi G. (2022). Inflammasome involvement in CS-induced damage in HaCaT keratinocytes. Vitr. Cell. Dev. Biol.-Anim..

[116] Awad F., Assrawi E., Louvrier C., Jumeau C., Giurgea I., Amselem S., Karabina S.A. (2018). Photoaging and skin cancer: Is the inflammasome the missing link?. Mech. Ageing Dev..

[117] Gurung P., Kanneganti T.D. (2016). Autoinflammatory Skin Disorders: The Inflammasomme in Focus. Trends Mol. Med..

[118] Vogeley C., Esser C., Tüting T., Krutmann J., Haarmann-Stemmann T. (2019). Role of the Aryl Hydrocarbon Receptor in Environmentally Induced Skin Aging and Skin Carcinogenesis. Int. J. Mol. Sci..

[119] Vogel C.F.A., Van Winkle L.S., Esser C., Haarmann-Stemmann T. (2020). The aryl hydrocarbon receptor as a target of environmental stressors—Implications for pollution mediated stress and inflammatory responses. Redox Biol..

[120] Neavin D.R., Liu D., Ray B., Weinshilboum R.M. (2018). The Role of the Aryl Hydrocarbon Receptor (AHR) in Immune and Inflammatory Diseases. Int. J. Mol. Sci..

[121] Afaq F., Abu Zaid M., Pelle E., Khan N., Syed D.N., Matsui M.S., Maes D., Mukhtar H. (2009). Aryl Hydrocarbon Receptor Is an Ozone Sensor in Human Skin. J. Investig. Dermatol..

[122] Qiao Y., Li Q., Du H.-Y., Wang Q.-W., Huang Y., Liu W. (2017). Airborne polycyclic aromatic hydrocarbons trigger human skin cells aging through aryl hydrocarbon receptor. Biochem. Biophys. Res. Commun..

[123] Ono Y., Torii K., Fritsche E., Shintani Y., Nishida E., Nakamura M., Shirakata Y., Haarmann-Stemmann T., Abel J., Krutmann J. (2013). Role of the aryl hydrocarbon receptor in tobacco smoke extract-induced matrix metalloproteinase-1 expression. Exp. Dermatol..

[124] Liu H., Shi L., Giesy J.P., Yu H. (2019). Polychlorinated diphenyl sulfides can induce ROS and genotoxicity via the AhR-CYP1A1 pathway. Chemosphere.

[125] Ibrahim M., MacFarlane E.M., Matteo G., Hoyeck M.P., Rick K.R.C., Farokhi S., Copley C.M., O’Dwyer S., Bruin J.E. (2020). Functional cytochrome P450 1A enzymes are induced in mouse and human islets following pollutant exposure. Diabetologia.

[126] Nebert D.W., Dalton T.P., Okey A.B., Gonzalez F.J. (2004). Role of Aryl Hydrocarbon Receptor-mediated Induction of the CYP1 Enzymes in Environmental Toxicity and Cancer. J. Biol. Chem..

[127] Guan L., Lim H., Mohammad T.F. (2021). Sunscreens and photoaging: A review of current literature. Am. J. Clin. Dermatol..

[128] Haywood R., Wardman P., Sanders R., Linge C. (2003). Sunscreens inadequately protect against ultraviolet-A-induced free radicals in skin: Implications for skin aging and melanoma?. J. Investig. Dermatol..

[129] Dumbuya H., Grimes P., Lynch S., Ji K., Brahmachary M., Zheng Q., Bouez C., Wangari-Talbot J. (2020). Impact of iron-oxide containiing formulations agasint visible light-induced skin pigmentation in skin of color individuals. J. Drug. Dermatol..

[130] Richard F., Creusot T., Catoire S., Egles C., Ficheux H. (2019). Mechanisms of pollutant-induced toxicity in skin and detoxification: Anti-pollution strategies and perspectives for cosmetic products. Ann. Pharm. Fr..

[131] Cao C., Xiao Z., Wu Y., Ge C. (2020). Diet and Skin Aging—From the Perspective of Food Nutrition. Nutrients.

[132] Ighodaro O.M., Akinloye O.A. (2018). First line defence antioxidants-superoxide dismutase (SOD), catalase (CAT) and glutathione peroxidase (GPX): Their fundamental role in the entire antioxidant defence grid. Alex. J. Med..

[133] Kohen R. (1999). Skin antioxidants: Their role in aging and in oxidative stress—New approaches for their evaluation. Biomed. Pharmacother..

[134] Shindo Y., Witt E., Han D., Epstein W., Packer L. (1994). Enzymic and Non-Enzymic Antioxidants in Epidermis and Dermis of Human Skin. J. Investig. Dermatol..

[135] Culotta V.C., Yang M., O’Halloran T.V. (2006). Activation of superoxide dismutases: Putting the metal to the pedal. Biochim. Biophys. Acta.

[136] Findlay G.H. (1963). Catalase Activity in Human Epidermis. Br. J. Dermatol..

[137] Wagener F.A., Carels C.E., Lundvig D. (2013). Targeting the redox balance in inflammatory skin conditions. Int. J. Mol. Sci..

[138] Kökçam I., Naziroǧlu M. (1999). Antioxidants and lipid peroxidation status in the blood of patients with psoriasis. Clin. Chim. Acta.

[139] Drewa G., Krzyzyńska-Malinowska E., Woźniak A., Protas-Drozd F., Mila-Kierzenkowska C., Rozwodowska M., Kowaliszyn B., Czajkowski R. (2002). Activity of superoxide dismutase and catalase and the level of lipid peroxidation products reactive with TBA in patients with psoriasis. Med Sci. Monit..

[140] Romani A., Cervellati C., Muresan X.M., Belmonte G., Pecorelli A., Cervellati F., Benedusi M., Evelson P.A., Valacchi G. (2018). Keratinocytes oxidative damage mechanisms related to airbone particle matter exposure. Mech. Ageing Dev..

[141] Treiber N., Maity P., Singh K., Ferchiu F., Wlaschek M., Scharffetter-Kochanek K. (2012). The role of manganese superoxide dismutase in skin aging. Dermato-Endocrinology.

[142] Shin M.H., Rhie G.-E., Kim Y.K., Park C.-H., Cho K.H., Kim K.H., Eun H.C., Chung J.H. (2005). H2O2 Accumulation by Catalase Reduction Changes MAP Kinase Signaling in Aged Human Skin In Vivo. J. Investig. Dermatol..

[143] Dijkhoff I.M., Drasler B., Karakocak B.B., Petri-Fink A., Valacchi G., Eeman M., Rothen-Rutishauser B. (2020). Impact of airborne particulate matter on skin: A systematic review from epidemiology to in vitro studies. Part. Fibre Toxicol..

[144] Syed D.N., Mukhtar H. (2012). Gender Bias in Skin Cancer: Role of Catalase Revealed. J. Investig. Dermatol..

[145] Niwa Y., Sumi H., Kawahira K., Terashima T., Nakamura T., Akamatsu H. (2003). Protein oxidative damage in the stratum corneum: Evidence for a link between environmental oxidants and the changing prevalence and nature of atopic dermatitis in Japan. Br. J. Dermatol..

[146] Valacchi G., Sticozzi C., Belmonte G., Cervellati F., Demaude J., Chen N., Krol Y., Oresajo C. (2015). Vitamin C Compound Mixtures Prevent Ozone-Induced Oxidative Damage in Human Keratinocytes as Initial Assessment of Pollution Protection. PLoS ONE.

[147] Valacchi G., Muresan X.M., Sticozzi C., Belmonte G., Pecorelli A., Cervellati F., Demaude J., Krol Y., Oresajo C. (2016). Ozone-induced damage in 3D-Skin Model is prevented by topical vitamin C and vitamin E compound mixtures application. J. Dermatol. Sci..

[148] Sticozzi C., Cervellati F., Muresan X.M., Cervellati C., Valacchi G. (2014). Resveratrol prevents cigarette smoke-induced keratinocytes damage. Food Funct..

[149] Ravetti S., Clemente C., Brignone S., Hergert L., Allemandi D., Palma S. (2019). Ascorbic Acid in Skin Health. Cosmetics.

[150] Thiele J.J., Ekanayake-Mudiyanselage S. (2007). Vitamin E in human skin: Organ-specific physiology and considerations for its use in dermatology. Mol. Aspects Med..

[151] Al-Niami F., Yi Zhen Chiang N. (2017). Topical Vitamin C and the Skin. J. Clin. Aesthethetic Dermatol..

[152] Burke K. (2007). Interaction of vitamins C and E as better cosmeceuticals. Dermatol. Ther..

[153] Lin J.-Y., Selim M., Shea C.R., Grichnik J.M., Omar M.M., Monteiro-Riviere N., Pinnell S.R. (2003). UV photoprotection by combination topical antioxidants vitamin C and vitamin E. J. Am. Acad. Dermatol..

[154] Thiele J.J., Traber M.G., Packer L. (1998). Depletion of Human Stratum Corneum Vitamin E: An Early and Sensitive In Vivo Marker of UV Induced Photo-Oxidation. J. Investig. Dermatol..

[155] Thiele J.J., Traber M.G., Tsang K., Cross C.E., Packer L. (1997). In Vivo Exposure to Ozone Depletes Vitamins C and E and Induces Lipid Peroxidation in Epidermal Layers of Murine Skin. Free Radic. Biol. Med..

[156] Thiele J.J., Traber M.G., Polefka T.G., Cross C.E., Packer L. (1997). Ozone-Exposure Depletes Vitamin E and Induces Lipid Peroxidation in Murine Stratum Corneum. J. Investig. Dermatol..

[157] Fahrenholtz S.R., Doleiden F.H., Trozzolo A.M., Lamola A.A. (1974). On the Quenching of Singlet Oxygen by α-Tocopherol. Photochem. Photobiol..

[158] Sguizzato M., Mariani P., Ferrara F., Drechsler M., Hallan S.S., Huang N., Simelière F., Khunti N., Cortesi R., Marchetti N. (2020). Nanoparticulate Gels for Cutaneous Administration of Caffeic Acid. Nanomaterials.

[159] Krol E.S., Kramer-Stickland K.A., Liebler D. (2000). Photoprotective Actions Of Topically Applied Vitamin E. Drug Metab. Rev..

[160] Chen L., Hu J.Y., Wang S.Q. (2012). The role of antioxidants in photoprotection: A critical review. J. Am. Acad. Dermatol..

[161] Chan A.C. (1993). Partners in defense, vitamin E and vitamin C. Can. J. Physiol. Pharmacol..

[162] Pinnell S.R., Yang H., Omar M., Monteiro-Riviere N., DeBuys H.V., Walker L.C., Wang Y., Levine M. (2001). Topical L-Ascorbic Acid: Percutaneous Absorption Studies. Dermatol. Surg..

[163] Lee S., Lee J., Choi Y.W. (2007). Skin Permeation Enhancement of Ascorbyl Palmitate by Liposomal Hydrogel (Lipogel) Formulation and Electrical Assistance. Biol. Pharm. Bull..

[164] Austria R., Semenzato A., Bettero A. (1997). Stability of vitamin C derivatives in solution and topical formulations. J. Pharm. Biomed. Anal..

[165] Lin F.-H., Lin J.-Y., Gupta R.D., Tournas J.A., Burch J.A., Selim M.A., Monteiro-Riviere N.A., Grichnik J.M., Zielinski J., Pinnell S.R. (2005). Ferulic Acid Stabilizes a Solution of Vitamins C and E and Doubles its Photoprotection of Skin. J. Investig. Dermatol..

[166] Murray J.C., Burch J.A., Streilein R.D., Iannacchione M.A., Hall R.P., Pinnell S.R. (2008). A topical antioxidant solution containing vitamins C and E stabilized by ferulic acid provides protection for human skin against damage caused by ultraviolet irradiation. J. Am. Acad. Dermatol..

[167] Oresajo C., Stephens T., Hino P.D., Law R.M., Yatskayer M., Foltis P., Pillai S., Pinnell S.R. (2008). Protective effects of a topical antioxidant mixture containing vitamin C, ferulic acid, and phloretin against ultraviolet-induced photodamage in human skin. J. Cosmet. Dermatol..

[168] Peres D.D., Sarruf F.D., de Oliveira C.A., Velasco M.V.R., Baby A.R. (2018). Ferulic acid photoprotective properties in association with UV filters: Multifunctional sunscreen with improved SPF and UVA-PF. J. Photochem. Photobiol. B Biol..

[169] Stahl W., Sies H. (2012). β-Carotene and other carotenoids in protection from sunlight. Am. J. Clin. Nutr..

[170] Anstey A.V. (2002). Systemic photoprotection with α-tocopherol (vitamin E) and β-carotene. Clin. Exp. Dermatol..

[171] Mitchell P. (1975). Protonmotive redox mechanism of the cytochrome *b-c* _1_ complex in the respiratory chain: Protonmotive ubiquinone cycle. FEBS Lett..

[172] Schreck R., Rieber P., Baeuerle P.A. (2010). Reactive oxygen intermediates as apparently widely used messengers in the activation of the NF-xB transcription factor and HIV-1. Biochem. Biophys. Res. Commun..

[173] Žmitek K., Pogačnik T., Mervic L., Žmitek J., Pravst I. (2017). The effect of dietary intake of coenzyme Q10 on skin parameters and condition: Results of a randomised, placebo-controlled, double-blind study: The Effect of Dietary Intake of Coenzyme Q10 on Skin Parameters and Condition. BioFactors.

[174] Knott A., Achterberg V., Smuda C., Mielke H., Sperling G., Dunckelmann K., Vogelsang A., Krüger A., Schwengler H., Behtash M. (2015). Topical treatment with coenzyme Q10-containing formulas improves skin’s Q10 level and provides antioxidative effects. BioFactors.

[175] El-Leithy E.S., Makky A.M., Khattab A.M., Hussein D.G. (2018). Optimization of nutraceutical coenzyme Q10 nanoemulsion with improved skin permeability and anti-wrinkle efficiency. Drug Dev. Ind. Pharm..

[176] Grether-Beck S., Marini A., Jaenicke T., Krutmann J. (2015). Effective photoprotection of human skin against infrared A radiation: Results from a vehicle controlled, randomized, double-blind study. Photochem. Photobiol..

[177] Grether-Beck S., Marini A., Jaenicke T., Krutmann J. (2014). Photoprotection of human skin beyond ultraviolet radiation. Photodermatol. Photoimmunol. Photomed..

[178] Lim H., Kohli I., Rubolo E., Kolbe L., Hamzavi I. (2022). Impact of visible light on skin health: The role of antioxidants and free radical quenchers in skin protection. J. Am. Acad. Dermatol..

[179] Hsu P.-Y., Yen H.-H., Yang T.-H., Su C.-C. (2018). Tetrathiomolybdate, a copper chelator inhibited imiquimod-induced skin inflammation in mice. J. Dermatol. Sci..

[180] Ghio A.J., Soukup J.M., Dailey L.A. (2016). Air pollution particles and iron homeostasis. Biochim. Biophys. Acta (BBA)-Gen. Subj..

[181] Ghio A.J., Soukup J.M., Dailey L.A., Madden M.C. (2020). Air pollutants disrupt iron homeostasis to impact oxidant generation, biological effects, and tissue injury. Free Radic. Biol. Med..

[182] Wright J.A., Richards T., Srai S.K.S. (2014). The role of iron in the skin and cutaneous wound healing. Front. Pharmacol..

[183] Reelfs O., MEggleston I., Pourzand C. (2010). Skin Protection Against UVA-Induced Iron Damage by Multiantioxidants and Iron Chelating Drugs/Prodrugs. Curr. Drug Metab..

[184] Welch K.D., Davis T., E Van Eden M., Aust S.D. (2002). Deleterious iron-mediated oxidation of biomolecules. Free Radic. Biol. Med..

[185] Hatcher H.C., Singh R.N., Torti F.M., Torti S.V. (2009). Synthetic and natural iron chelators: Therapeutic potential and clinical use. Future Med. Chem..

[186] Mobarra N., Shanaki M., Ehteram H., Nasiri H., Sahmani M., Saeidi M., Goudarzi M., Pourkarim H., Azad M. (2016). A Review on Iron Chelators in Treatment of Iron Overload Syndromes. Int. J. Hematol. Stem Cell Res..

[187] Dev S., Babitt J.L. (2018). Overview of iron metabolism in health and disease. Hemodial. Int..

[188] Applegate L.A., Noël A., Vile G., Frenk E., Tyrrell R.M. (1995). Two Genes Contribute to Different Extents to the Heme Oxygenase Enzyme Activity Measured in Cultured Human Skin Fibroblasts and Keratinocytes: Implications for Protection Against Oxidant Stress. Photochem. Photobiol..

[189] Schreinemachers D.M., Ghio A.J. (2016). Article Commentary: Effects of Environmental Pollutants on Cellular Iron Homeostasis and Ultimate Links to Human Disease. Environ. Health Insights.

[190] Bissett D.L., McBride J.F. (1996). Synergistic topical photoprotection by a combination of the iron chelator 2-furildioxime and sunscreen. J. Am. Acad. Dermatol..

[191] Mitani H., Koshiishi I., Sumita T., Imanari T. (2001). Prevention of the photodamage in the hairless mouse dorsal skin by kojic acid as an iron chelator. Eur. J. Pharmacol..

[192] Shalev O., Hebbel R. (1996). Extremely high avidity association of Fe(III) with the sickle red cell membrane. Blood.

[193] Franchini M., Gandini G., Veneri D., Aprili G. (2004). Safety and efficacy of subcutaneous bolus injection of deferoxamine in adult patients with iron overload: An update. Blood.

[194] Hou Z., Nie C., Si Z., Ma Y. (2013). Deferoxamine enhances neovascularization and accelerates wound healing in diabetic rats via the accumulation of hypoxia-inducible factor-1α. Diabetes Res. Clin. Pract..

[195] Duscher D., Neofytou E., Wong V.W., Maan Z.N., Rennert R.C., Inayathullah M., Januszyk M., Rodrigues M., Malkovskiy A.V., Whitmore A.J. (2015). Transdermal deferoxamine prevents pressure-induced diabetic ulcers. Proc. Natl. Acad. Sci. USA.

[196] Duscher D., Trotsyuk A.A., Maan Z.N., Kwon S.H., Rodrigues M., Engel K., Stern-Buchbinder Z.A., Bonham C.A., Barrera J., Whittam A.J. (2019). Optimization of transdermal deferoxamine leads to enhanced efficacy in healing skin wounds. J. Control. Release.

[197] Kong L., Wu Z., Zhao H., Cui H., Shen J., Chang J., Li H., He Y. (2018). Bioactive Injectable Hydrogels Containing Desferrioxamine and Bioglass for Diabetic Wound Healing. ACS Appl. Mater. Interfaces.

[198] Davies M.J., Donkor R., Dunster C.A., Gee C.A., Jonas S., Willson R.L. (1987). Desferrioxamine (Desferal) and superoxide free radicals. Formation of an enzyme-damaging nitroxide. Biochem. J..

[199] Parvar M., Mehrzad J., Chaichi M.J., Hosseinkhani S., Golchoubian H. (2014). Quenching effect of deferoxamine on free radical-mediated photon production in luminol and ortho-phenanthroline-dependent chemiluminescence. Chin. Chem. Lett..

[200] Hartley A., Davies M., Rice-Evans C. (1990). Desferrioxamine as a lipid chain-breaking antioxidant in sickle erythrocyte membranes. FEBS Lett..

[201] Pourzand C., Albieri-Borges A., Raczek N. (2022). Shedding a new light on skin aging, iron and redox-homeostasis and emerging natural antioxidants. Antioxidants.

[202] Michalak M. (2022). Plant-Derived Antioxidants: Significance in Skin Health and the Ageing Process. Int. J. Mol. Sci..

[203] Luze H., Nischwitz S.P., Zalaudek I., Mulleger R., Kamolz L.P. (2020). DNA repair enzymes in sunscreens and their impact on photoageing- A systemic review. Photodermatol. Photoimmunol. Photomed..

[204] Kern J., Wood E., Almukhtar R., Angra K., Lipp M., Goldman M. (2022). Evaluation of an SPF50 sunscreen containing photolyase and antioxidants for its anti-photoaging and photoprotection. J. Drugs Dermatol. JDD.

[205] Yarosh D.B., Rosenthal A., Moy R. (2019). Six critical questions for DNA repair enzymes in skincare products: A review in dialog. Clin. Cosmet. Investig. Dermatol..

[206] Bhatia N., Berman B., Ceilley R.I., Kircik L.H. (2017). Understanding the Role of Photolyases: Photoprotection and Beyond. J. Drugs Dermatol..

[207] Kabir Y., Seidel R., McKnight B., Moy R. (2015). DNA repair enzymes: An important role in skin cancer prevention and reversal of photodamage—A review of the literature. J. Drugs Dermatol..

[208] Puviani M., Barcella A., Milani M. (2013). Efficacy of a photolyase-based device in the treatment of cancerization field in patients with acticinic keratosis and non-melanoma skin cancer. G. Ital. Dermatol. Venereol..

